# Simultaneous occurrence of IgG4-related Tubulointerstitial nephritis and colon adenocarcinoma with hepatic metastasis: a case report and literature review

**DOI:** 10.1186/s12882-019-1205-5

**Published:** 2019-01-16

**Authors:** Shen-Ju Gou, Lu-Jia Xue, Zhang-Xue Hu

**Affiliations:** Department of Nephrology, West China Hospital, Sichuan University, Chengdu, 610041 China

**Keywords:** IgG4-related disease, Malignancy, Metastasis, Renal dysfunction

## Abstract

**Background:**

Understanding the uncommon association of IgG4-related disease with other disorders is essential for the accurate diagnosis and effective treatment of patients. To the best of our knowledge, there have been only few reports of patients with IgG4-related kidney disease coexisting with metastasis of malignancy. Here, we report a rare case of simultaneous occurring IgG4-related tubulointerstitial nephritis and colon adenocarcinoma with hepatic metastasis.

**Case presentation:**

A 71-year-old Chinese man presented with dysuria and was initially diagnosed as benign prostatic hyperplasia for one year. He was admitted to the hospital for surgery. After admission, the renal function tests revealed a rapid increase of serum creatinine from 291.0 μmol/L to 415 μmol/L. The hemoglobin level was 89 g/L. Fecal occult blood testing was positive. Urinalysis revealed mild proteinuria. The serum IgG4 level was 13.9 g/L. The abdominal imaging examination revealed multiple solid nodules in the liver. The gastrointestinal endoscopy combined with the biopsy revealed colon adenocarcinoma. Kidney biopsy showed massive IgG4-positive plasma cells and storiform fibrosis infiltration in the tubulointerstitial area, thus establishing the diagnosis of IgG4-related tubulointerstitial nephritis. Corticosteroid therapy was initiated, and subsequently, the renal function dramatically improved without the diminution of the liver nodules. The liver biopsy was performed and a diagnosis of metastatic colon adenocarcinoma was confirmed.

**Conclusions:**

We here reported a rare case of simultaneous occurring of IgG4-related tubulointerstitial nephritis, colon adenocarcinoma with hepatic metastasis. The case highlights the importance of screening for malignancy in patients with IgG4-related disease, and the nature of the mass in other organs of patients with coexisting IgG4-related disease and malignancy should be carefully checked.

## Background

Immunoglobulin G4-related disease (IgG4-RD) is an immune-mediated systemic disorder which may involve potentially every organ or system [1]. The pathological hallmarks of IgG4-RD are substantial infiltration of IgG4-positive plasma cells in tissues and storiform fibrosis. Given its tendency to occur as mass lesions in some organs, IgG4-RD is often confused as malignancy. In addition, recent studies had suggested an association between IgG4-related disease and the malignancies [2–5]. However, the malignancies reported in the previous studies were mostly primary malignancies, and the IgG4-RD usually involved multiple organs. To the best of our knowledge, coexisting of metastatic malignancy and IgG4-related disease with kidney involvement was rare. Here, we report a rare case of simultaneous occurring of IgG4-related tubulointerstitial nephritis and colon adenocarcinoma with hepatic metastasis.

## Case presentation

**Fig. 1:**
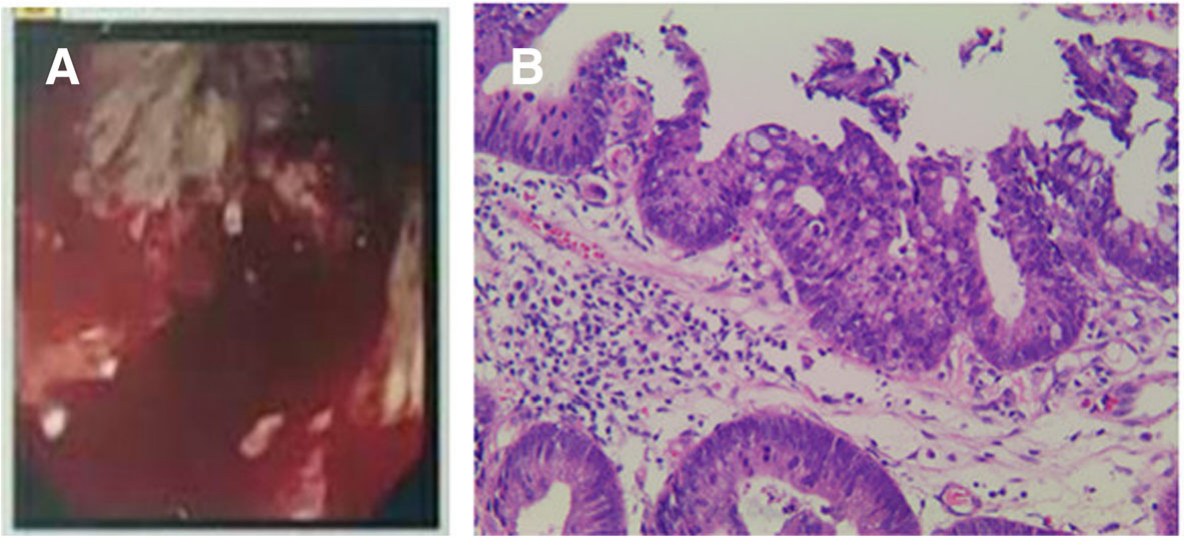
The result of colonoscopy and histopathological findings of the colon specimen. (**a**) The gastrointestinal endoscopy result showed a circular cauliflower shaped, ulcerative mass at the middle section of the transverse colon. (**b**) Biopsies of the colonic mass revealed adenocarcinoma (amplification × 200)

A 71-year-old Chinese man presented with urinary hesitancy, dribbling urination, and prolonged urination and was diagnosed as benign prostatic hyperplasia at out-patient one year ago. The serum creatinine was 101 μmol/L (normal range 53~140 μmol/L) at that moment. He was prescribed with epristeride and tamsulosin. Nine months ago, the patient stopped the oral medication because of loss of appetite. The symptoms of urinary hesitancy, dribbling and prolonged urination worsened gradually and therefore he was admitted to our hospital for surgery. On admission, the renal function test revealed a serum creatinine level of 291.0 μmol/L. The post-void residual was normal. The ultrasonic examination revealed that both kidneys were normal in structure and size (left 11.6 cm × 6.3 cm,right 10.7 cm × 4.4 cm). Obstructive nephropathy was thus excluded and the surgery was canceled for renal dysfunction. The patient was transferred to renal division of internal medicine department where additional tests were performed in order to establish the etiology of his documented renal failure. The results of routine peripheral blood test were as follows: hemoglobin 89 g/L (normal range 130~175 g/L), white blood cells 5.21 × 10^9^/L (normal range 3.5~9.55.21 × 10^9^/L), and platelets 204 × 10^9^/L (normal range 100~300 × 10^9^/L). Urinalysis was positive for 1+ protein. Red blood cells and white blood cells were negative in urine sediment microscopic examination. The 24 h urinary protein determination was 0.67 g. Fecal occult blood testing was positive. In addition, the serum creatinine level increased to 415 μmol/L. The immunology tests revealed the following: anti-nuclear antibody + 1:100, rheumatoid factor 149 IU/ml (normal range < 20 IU/ml), IgG 23 g/L (normal range 8~15.5 g/L), serum IgG4 13.9 g/L (normal range 0.035~1.5 g/L), IgE 288.7 IU/ml (normal range 0.1~150 IU/ml), C3 0.4310 g/L (normal range 0.785~1.520 g/L), C4 0.0362 g/L (normal range 0.145~0.360 g/L). The direct Coomb’s test was negative. The anti-neutrophil cytoplasmic antibodies and anti-glomerular basement membrane antibody were both negative. The abdominal ultrasonography revealed multiple solid nodules in the liver. Magnetic resonance imaging (MRI) confirmed multiple liver parenchymal round shaped long T1 and long T2 signal nodules, with a diameter of between 0.6 and 16 cm. The nodules revealed mild enhancement during arterial enhancement phase with some of them showed a decline of enhancement during portal enhancement period. Since the patient has gastrointestinal symptoms in combination with positive fecal occult blood test and moderate anemia, a gastrointestinal endoscopy was performed and It showed a circular cauliflower shaped, ulcerative mass at the middle section of the transverse colon. Biopsies of the mass revealed adenocarcinoma (Fig. 1).

**Fig. 2:**
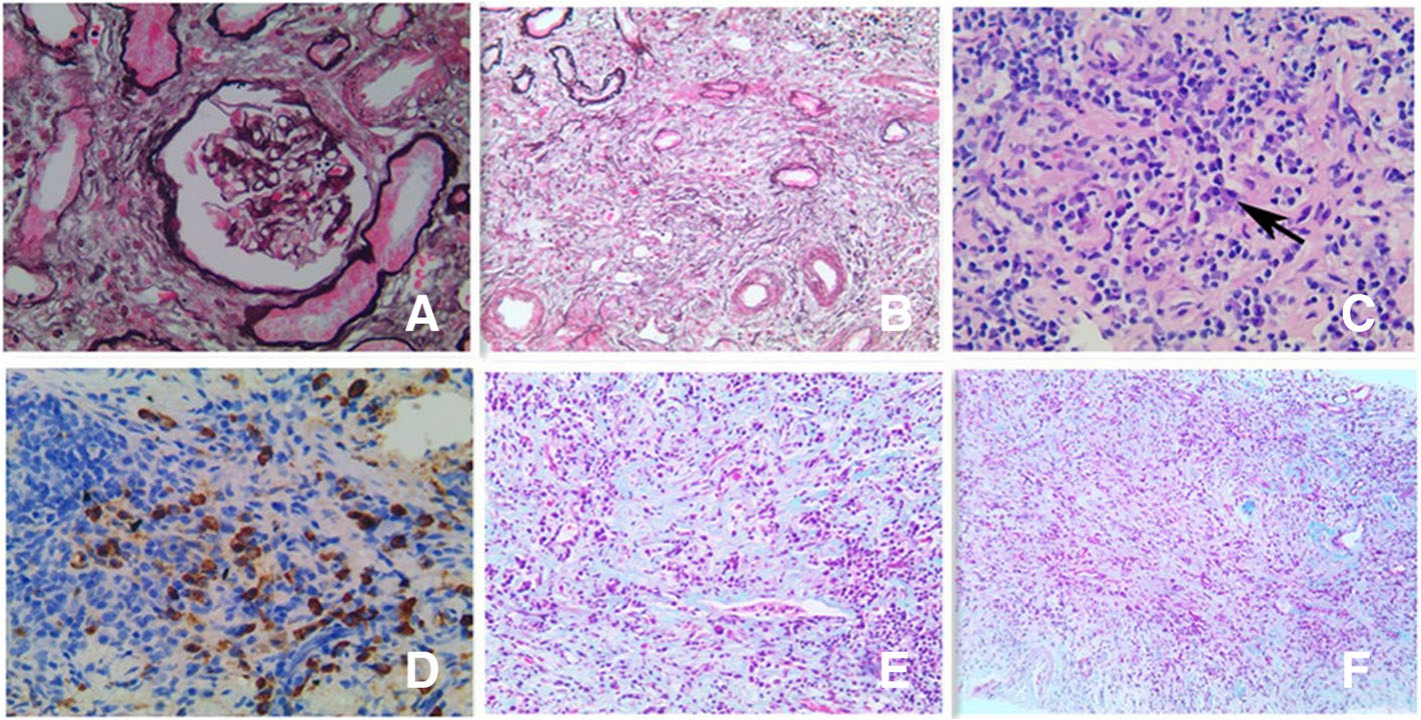
The histopathological and immunohistochemical findings of the renal specimen. (**a**) Periodic acid-silver metheramine staining showed mild injury in the glomeruli (amplification × 400). (**b**) Periodic acid-silver metheramine staining showed storiform fibrosis in the tubulointerstitial compartment (amplification × 200). (**c**) Haematoxylin and eosin staining showed massive lymphocytes and plasma cells infiltration in the tubulointerstitial compartment (amplification × 400). The black arrow indicated a plasma cell. (**d**) The immunohistochemistry staining showed IgG4-positive plasma cells infiltration in the interstitial compartment (amplification × 400). (**e**) Masson’s trichrome staining showed storiform fibrosis in the tubulointerstitial compartment (amplification × 200). (**f**) Masson’s trichrome staining showed massive inflammatory cells infiltration and fibrosis in the tubulointerstitial compartment (amplification × 100)

For evaluation of renal dysfunction, a renal biopsy was performed. The pathological findings in light microscopy demonstrated glomerular sclerosis in two of twelve glomeruli whereas the other glomeruli demonstrated only mild lesions. The periodic acid-silver metheramine and Masson’s trichrome stainings showed 75% interstitial fibrosis and tubular atrophy in the tubulointerstitial area. In the fibrotic interstitial compartment, collagen fibers exhibited a storiform pattern, with massive lymphocyte and plasma cells infiltration. Immunohistochemical staining showed more than 30 IgG4-positive plasma cells per high-power field (Fig. 2a-f). Immunofluorescence testing was negative for IgG, IgA, IgM, C3, C4, C1q, κ chain, and λ chains in glomeruli. A diagnosis of IgG4-related tubulointerstitial nephritis (IgG4-TIN) was thus made.

**Fig. 3:**
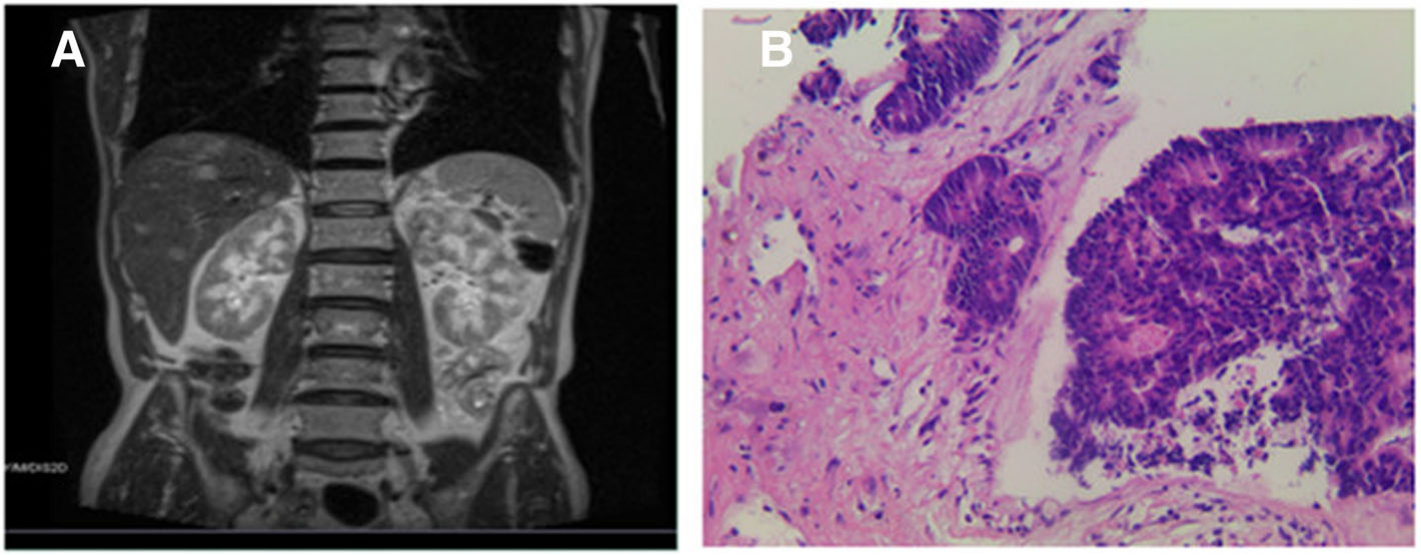
The magnetic resonance imaging of the abdomen and the histopathological finding of the liver specimen. (**a**) The magnetic resonance imaging revealed multiple nodules in the liver. (**b**) The pathology of the hepatic nodule revealed adenocarcinoma (amplification × 200)

**Fig. 4:**
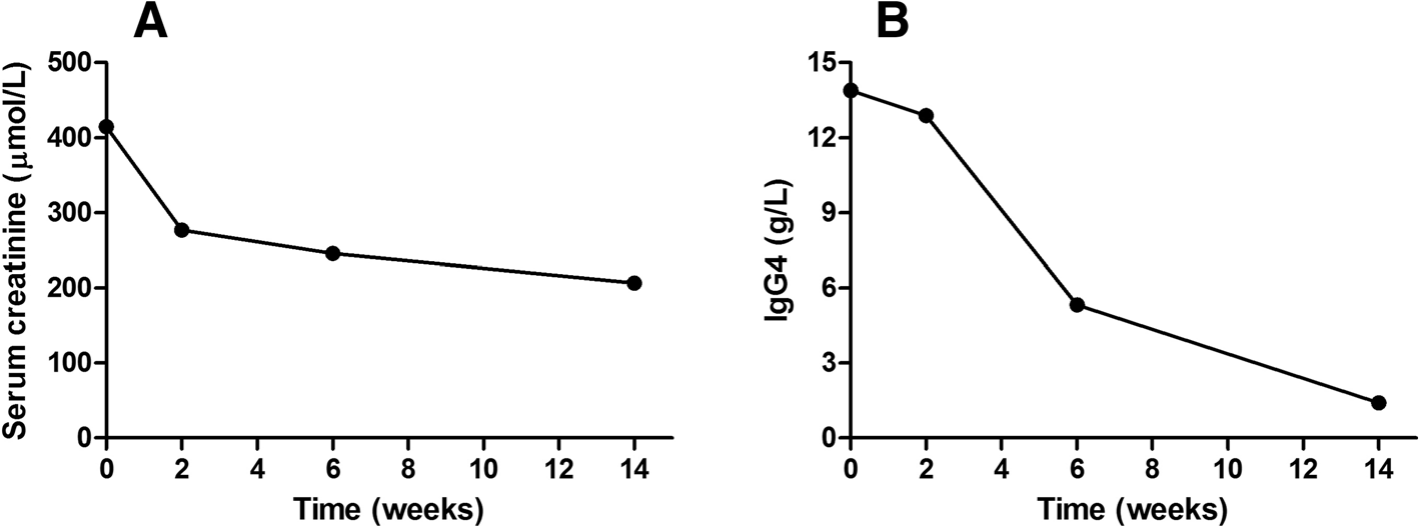
The clinical course of the patient after corticosteroid treatment was initiated. (**a**) The changes of serum creatinine. (**b**) The changes of serum IgG4

As previously established, both IgG4-related disease and metastasis of gastrointestinal tumor could cause hepatic occupying lesions. In that sense, liver nodules in the current case could be secondary to either IgG4-related tubulointerstitial nephritis or remote metastasis from colon adenocarcinoma. Prednisone of 1 mg/kg daily was initiated with the objective to treat IgG4-TIN. On the one hand, the treatment might improve renal function, the improvement of renal function would then create better conditions for chemotherapy or surgery of adenocarcinoma treatment. On the other hand, the imaging response of hepatic nodules to glucocorticoid administration might suggest whether the nodules were malignancy or IgG4-related pseudo-tumor. One and a half month later, the serum creatinine had decreased from 415 to 246 μmol/L, and the serum IgG4 level dropped from 13.9 g/L to 5.3 g/L. However, the repeat MRI revealed no diminution of hepatic nodules. A liver biopsy was performed and atypical glands were founded in the specimen (Fig. 3). Based on the findings of immunohistochemistry of the specimen and clinical data, a diagnosis of adenocarcinoma with hepatic metastasis was made. Chemotherapy was recommended by the Oncology team although impaired renal function was a contraindication. Prednisone was continued to improve kidney function in order to propitiate conditions for chemotherapy administration. Prednisone was gradually tapered and 14 weeks later, the serum creatinine level was 207 μmol/L and the serum IgG4 level was 1.41 g/L (Fig. 4). Unfortunately, five months later, the patient’s general condition deteriorated quickly. The patient suffered from anorexia and poor mental state. During the last follow-up, occurring half-year later, the patient experienced shortness of breath but refused to be admitted and died two days later. The last serum creatinine level tested was 176 μmol/L.

## Discussion and conclusions

IgG4-RD is a newly recognized fibroinflammatory condition that often causes tumefactive lesions and has the potential to affect any organ in the body. Histologically, IgG4-RD is characterized by dense lymphoplasmacytic infiltration with abundant IgG4-positive plasma cells, storiform fibrosis formation, and obliterative phlebitis. The disease is usually manifested as enlargement or hyperplasia of involved organs and therefore it can be easily mistaken as malignancy. In addition, recent studies had suggested an association between IgG4-related disease and malignancies [2–5]. As treatment and prognosis of patients with IgG4-RD are totally different from patients with malignancies, an accurate diagnosis is crucial in clinical practice. In the present case, the patients was diagnosed as IgG4-TIN in combination with colon adenocarcinoma, both demonstrated pathologically. In addition, abdominal ultrasound and MRI revealed multiple hepatic nodules. Previous studies have showed that hepatic inflammatory pseudo-tumors or tumefactive nodules are possible manifestations of intrahepatic IgG4-related sclerosing cholangitis [6], thus suggesting that both IgG4-RD and adenocarcinoma could cause the hepatic nodules observed in this case. The etiology of the hepatic nodules would dramatically influence the treatment whether the patient should be operated or not. Previous reports concluded that it could be difficult to differentiate IgG4-RD from malignancy by using MRI or computed tomography (CT) [7]. Even the positron emission tomography and computed tomography (PET/CT) could not accurately distinguish IgG4-RD from malignancy as both diseases can be associated with an increased ^18^F-FDG uptake [8]. As it is difficult to discriminate both diseases by radiology alone, tissue biopsy emerge as be the best option for establishing the nature of tumor-like swelling or masses in patients with coexisting IgG4-RD and malignancy.

A retrospective study of 166 patients with IgG4-RD (January 1994 to September 2012), conducted by Sekiguchi et al., reported that 10% of the patients (17/166) had a history of malignancy before the diagnosis of IgG4-RD, and 5% of the patients received a diagnosis of malignancy after the diagnosis of IgG4-RD [4]. IgG4-RD involved potentially every organ or system, occasionally including kidney. It was reported that nearly 30% of the IgG4-RD might have tubulointerstitial nephritis. The association of IgG4-TIN and malignancy was described in several isolated case reports [9–13], which was summarized in Table 1. However, the malignancies reported in the previous studies were mostly primary malignancies. To the best of our knowledge, metastasis of malignancy occurred simultaneously with IgG4-TIN was rare. Here, we report a rare case of simultaneous occurring of IgG4-TIN and colon adenocarcinoma with hepatic metastasis.

**Table 1:** Previous reports of concurrence of IgG4-related tubulointerstitial nephritis and malignancy

Reported by	Gender	Age	Malignancy	Treatment of malignancy	Time to diagnose IgG4-TIN	Metastasis of malignancy before the diagnosis of IgG4-TIN
Horita S	male	81	gastric cancer	gastrectomy	6 months later after the diagnosis of malignancy	No
Watanabe R	male	61	renal cell carcinoma	segmental resection of the kidney	2 years later after the diagnosis of malignancy	No
Oshima Y	male	41	follicular cell lymphoma	surgical resection and chemotherapy	14 years later after the diagnosis of malignancy	No
Krebs S	female	64	breast carcinoma	detailed therapy was not reported	3 years later after the diagnosis of malignancy	No
Takashi M	male	68	lung cancer	right lung lobectomy and chemotherapy	15 months later after the malignancy	metastases to cerebellum, hilar lymph nodes, and adrenal gland

In previous studies, there have been several reports regarding malignancies in IgG4-RD patients. In American population-based studies by Wallace et al. [14] and Sekiguchi et al. [4], prostate cancer was the most common malignancy associated with IgG4-RD. In Japanese population-based study by Hirano et al., lung cancer was the most common malignancy associated with IgG4-RD. In Korean population-based study by Ahn et al., lymphoma was the most frequent malignancy associated with IgG4-RD [15]. In Chinese population-based study by Feng et al., colorectal cancer was the most frequent malignancy associated with IgG4-RD [16]. The primary organ with malignancy in the present case report was in line with the Chinese data. Since the malignancies associated with IgG4-RD patients have different organ priorities in different population, screening for malignancy targeting key organs should be individualized in different countries.

Recent studies identified a close association between IgG4-RD and malignancy. The standardized incidence ratio (SIR) of malignancies in IgG4-RD was reported to be higher than that in general population [5, 17, 18]. It was inferred that a chronic inflammatory state caused by IgG4-RD might play a role in malignancy development [15]. However, it is interesting that the SIR of patients who exhibited a tumor within 1 year after IgG4-RD diagnosis was higher than general population, but the SIR of patients forming a malignancy after one year was not significantly higher than general population [17]. Furthermore, in order to clarify whether IgG4-RD could cause malignancy, the incidence of malignancy in 113 patients with IgG4-RD during a long-term follow-up period was studied by Hirano et al. [2]. It was found that if excluding the patients in whom malignancy was diagnosed ≤6 months before or after the onset of IgG4-RD and those whose follow-up period was≤6 months, the incidence of malignancies in IgG4-RD patients was similar to that of the general population, thus concluding that IgG4-RD was not associated with an increased incidence of malignancies. On the other side, recent studies had frequently reported IgG4-RD patients having a history of malignancy preceding the clinical onset of IgG4-RD. For example, Wallace et al. observed that as much as 16% of IgG4-RD patients had a previous diagnosis of malignancy before the diagnosis of IgG4-RD [14]. The observed prevalence of malignancy was significantly higher than in matched controls, which suggested that malignancy might be associated with subsequent IgG4-RD development in a subset of patients with IgG4-RD. It was speculated that autoantigen expression triggered by cancer might play a part in the development of IgG4-RD, and the cancer treatment might increase the risk of IgG4-RD development. The shared risk factors of both IgG4-RD and cancer might also contribute to the concurrence of the two diseases. However, Up to now, the patients with IgG4-RD might be diagnosed with a malignancy before the diagnosis of IgG4-RD, concurrently, or during follow-up. The definite mechanism of development of malignancy and IgG4-RD in one patient is not clear and needs further study.

In conclusion, we reported a rare case of simultaneous occurring of IgG4-TIN and colon adenocarcinoma with hepatic metastasis. It is important to screen for malignancy in patients with IgG4-RD, although the definite mechanism of concurrence of malignancy and IgG4-RD is not clear and needs further study. The nature of the organ masses in patients with coexisting IgG4-RD and malignancy should be carefully studied.

## Abbreviation

CT: computed tomography
IgG4-RD: Immunoglobulin G4-related disease
IgG4-TIN: IgG4-related tubulointerstitial nephritis
MRI: magnetic resonance imaging
PET/CT: positron emission tomography and computed tomography
SIR: Standardized incidence ratio
